# Amniotic Suspension Allograft for Treatment of Knee Osteoarthritis

**DOI:** 10.3390/biomedicines10102658

**Published:** 2022-10-21

**Authors:** Ashim Gupta

**Affiliations:** 1 Regenerative Orthopaedics, Noida 201301, UP, India; ashim6786@gmail.com; 2 Indian Stem Cell Study Group (ISCSG) Association, Lucknow 226010, UP, India; 3 Future Biologics, Noida 201301, UP, India; 4 BioIntegrate, Lawrenceville, GA 30043, USA; 5 South Texas Orthopaedic Research Institute (STORI Inc.), Laredo, TX 78045, USA

Osteoarthritis (OA) is an immensely pervasive joint disorder—typically concerning large weight-bearing joints—affecting over 30 million people in the United States, with this number predicted to reach 67 million by 2030 [1]. Its pathophysiology entails synovial tissue inflammation and articular cartilage degeneration, leading to pain and reduced function [1,2,3]. Mostly, OA is managed with physical therapy, activity adjustment, pharmacological agents (for example, non-steroidal anti-inflammatory drugs (NSAIDs), opioids, corticosteroids, viscosupplementation (hyaluronic acid), etc.), and surgery when well-known treatment modalities have failed [1]. The above-mentioned treatment approaches have constraints, seeking to decrease pain rather than targeting the inherent pathology [1].

Recently, various molecular targets, such as, interleukin-1 (IL-1), transforming growth factor-β (TGF-β), matrix metalloproteinases (MMPs), etc., have been reported to be linked in the etiopathogenesis of OA [4,5,6]; however, these therapies may well have a negative risk-to-benefit ratio [7,8]. Consequently, further safe and effective treatment alternatives are warranted to handle this unmet medical need.

Over the last decade, there has been a heightened interest in the use of biologics, including autologous biologics such as platelet-rich plasma (PRP), bone marrow concentrate, adipose tissue, and allogenic biologics, such as perinatal tissue for regenerative medicine applications specifically for musculoskeletal disorders [1]. Perinatal allogenic tissue includes amniotic tissue (amniotic membrane and/or amniotic fluid) and has been used clinically for several years for burns, complex wounds and ophthalmic conditions [9,10,11]. Lately, the utilization of amniotic tissue for musculoskeletal conditions, including plantar fasciitis, tendinopathies, cartilage defects, etc., has also grown [12]. Numerous basic science studies have demonstrated the presence of anti-inflammatory cytokines such as IL-1 receptor antagonist (IL-1RA), MMPs, hylauronic acid (HA) and proteoglycans in amniotic tissue, indicating a potential role in the treatment of OA [13,14]. Several preclinical studies, as discussed earlier [1], have shown positive outcomes in rat and rabbit OA models [15,16,17,18,19]. Despite these promising results, there are limited high-powered, clinical trials showing the safety and efficacy of amniotic tissue for the treatment of patients suffering with knee OA.

In this Editorial, I will focus on a recently published clinical trial by Natali et al. [20], titled, “Human Amniotic Suspension Allograft Improves Pain and Function in Knee Osteoarthritis: A Prospective Not Randomized Clinical Pilot Study”. In this prospective, non-randomized study, the authors investigated the safety, clinical effectiveness and feasibility of intra-articular injections of amniotic suspension allograft (ASA) in patients suffering with unilateral knee OA, with the aim of evaluating the improvement in clinical status and delaying any invasive surgical procedures. A total of 25 patients (11 males and 14 females) were enrolled in the study in line with inclusion (Kellgren–Lawrence (KL) grade 1-3, failure of prior conservative treatments, i.e., NSAIDs, physical therapy, intra-articular injections of corticosteroids, HA or PRP, etc.) and exclusion criteria (KL grade 4, intra-articular steroid or HA within last 3 months, etc.), and injected with 3 mL ASA (*homogenized amniotic membrane suspended in physiological solution*). These patients were assessed at baseline (prior to injection) and at 3, 6, and 12 months post-injection using the International Knee Documentation Committee (IKDC) and Visual Analogue Scale (VAS) scores. No severe adverse events were reported throughout the duration of the study. Statistically significant improvements (*p* < 0.05) were observed for both IKDC and VAS at all follow-up time points compared with the baseline. Interestingly, both IKDC and VAS scores regressed by 6 months, indicating a lack of long-lasting effect of ASA; however, at the 12-month follow-up, both scores indicated significant improvement compared to the baseline. In spite of this, the results from this study indicated that a single intra-articular injection of ASA is safe and showed positive clinical outcomes, which is in accordance with other published clinical trials utilizing ASA for the treatment of knee OA [21,22,23]. This study has a few limitations, as also indicated by the study’s authors. These include small sample size, lack of placebo and/or control group and analysis of MRI images.

In addition to the aforementioned limitations, one of the concerns, not limited to this study, is lack of consistency in the composition of similarly named biologics. For instance, this study used the term ASA and defined it as a ‘*homogenized amniotic membrane suspended in physiological solution*’, whereas previously published studies [21,22,23] defined ASA as ‘*amniotic suspension allograft that contains human amniotic membrane and human amniotic fluid-derived cells*’, with no description of the formulation protocol. Thus, I believe, it is essential to maintain uniformity in the composition of similarly named biologics and to describe the formulation protocol to allow the repeatability and reproducibility of the results of prospective trials assessing the safety and efficacy of these biologics throughout the world, in order to ultimately justify their clinical usage.

In summary, despite limitations, I applaud the efforts of the authors, as this study positively adds to the current literature suggesting that the administration of amniotic tissue including ASA is safe and justifies the need for a high-powered, prospective, multi-center, double-blind, randomized controlled trial with a longer follow-up duration to further establish the efficacy of ASA to alleviate symptoms associated with knee OA, thereby, possibly providing a new minimally invasive therapeutic alternative for patients suffering with knee OA. As of 13 October 2022, there are three ongoing clinical trials registered on clinicaltrials.gov (search terms: “knee osteoarthritis” and “amniotic suspension allograft” or ‘amniotic membrane”). These trials are summarized in Table 1.

## Figures and Tables

**Table 1:** Ongoing clinical trials registered on ClinicalTrials.gov as of 13 October 2022 utilizing amniotic suspension allograft (ASA) or amniotic membrane for the treatment of knee osteoarthritis.

Study Identifier	Biologic (*Description*)	Study Phase; Estimated Enrollment (N)	Primary Outcome Measure(s)	Recruitment Status	Country
NCT04636229	Amniotic suspension allograft (*amniotic suspension allograft that contains human amniotic membrane and human amniotic fluid-derived cells*)	Phase III; N = 474	The difference in change from baseline in WOMAC pain scale at 6 months between ASA- and placebo-treated patients (time frame: baseline to week 26). The Western Ontario and McMaster Universities (WOMAC^®®^) Osteoarthritis Index is a questionnaire that measures pain, stiffness, and function both independently and collectively, using a Likert 3.1, 5-point scale. The Likert Scale uses the following descriptors for all items: none, mild moderate, severe, and extreme, corresponding to an ordinal scale of 0–4. Higher scores on the WOMAC indicate worse pain, stiffness, and functional limitations.	Recruiting	USA
NCT04698265	Amniotic suspension allograft (*particulate human amniotic membrane and cells derived from amniotic fluid*)	Not applicable; N = 150	Change in the Western Ontario and McMaster Universities Arthritis Index (WOMAC) between baseline, 1 week, and 1, 3, 6, 12 months (time frame: baseline, 1 week, 1, 3, 6, 12 months). WOMAC is a self-administered questionnaire consisting of 24 items divided into 3 subscales: (1) pain (5 items): during walking, using stairs, in bed, sitting or lying, and standing upright; (2) stiffness (2 items): after first waking and later in the day; (3) physical function (17 items): using stairs, rising from sitting, standing, bending, walking, getting in/out of a car, shopping, putting on/taking off socks, rising from bed, lying in bed, getting in/out of bath, sitting, getting on/off toilet, heavy domestic duties, light domestic duties. The test questions are scored on a scale of 0–4, which correspond to: none (0), mild (1), moderate (2), severe (3), and extreme (4).	Not yet recruiting	Taiwan
NCT04612023	Amniotic membrane (*acellular amniotic membrane*)	Phase II; N = 90	Primary efficacy endpoints using validated patient-reported outcome tools questionnaires (time frame: 1 year). Knee injury and Osteoarthritis Outcome Score (KOOS) assessing five outcomes: pain, symptoms, activities of daily living, sport and recreation function, and knee-related quality of life. This is a 0–100 scale, with zero representing extreme knee problems and 100 representing no knee problems. Primary efficacy endpoints using validated patient-reported outcome tool questionnaires (time frame: 1 year). Western Ontario and McMaster Universities Osteoarthritis Index (WOMAC) assessing the condition of patients with osteoarthritis of the knee and hip, including pain, stiffness, and physical functioning of the joints. This method uses a 0 (worst)–96 scale (best). Primary efficacy endpoints using validated patient-reported outcome tool questionnaires (time frame: 1 year). The Visual Analogue Scale (VAS) assesses pain on a 0–100 scale. A higher score indicates greater pain intensity.	Recruiting	USA
